# Intratympanic corticosteroid injection for Ménière’s disease: systematic review of randomized controlled trials

**DOI:** 10.1007/s00405-025-09764-4

**Published:** 2025-11-15

**Authors:** Helena Guilera Martínez, Francesc Larrosa, Marta Sandoval, Joan Remacha, Manuel Bernal-Sprekelsen

**Affiliations:** 1 Medical School, Universitat de Barcelona, Barcelona, Spain; 2 Hospital Clinic, University of Barcelona, Barcelona, Spain; 3 Hospital Clinic, Barcelona, Spain; 4 Servicio de ORL Hospital Clinic, Villarroel 170, Esc.9, 2A, Barcelona, 08036 Spain; 5 University of Barcelona, Faculty of Medicine, Departament d´Especialitats Quirùrgiques, c/Casanova 143, 08036 Barcelona, Spain

**Keywords:** Ménière’s disease, Intratympanic corticosteroids, Dexamethasone, OTO-104, Vertigo, Tinnitus, Systematic review

## Abstract

**Introduction:**

Menière’s disease is a chronic inner ear disorder marked by episodic vertigo, tinnitus, fluctuating sensorineural hearing loss, and aural fullness, all of which significantly impair quality of life. Intratympanic corticosteroids (ITCs) have emerged as a potential therapy, but their clinical efficacy and safety remain uncertain.

**Methods:**

A systematic review was conducted of randomized controlled trials (RCTs) comparing ITC to placebo in adult MD patients. Comprehensive searches were performed across multiple electronic databases. Six RCTs met the inclusion criteria, evaluating formulations such as dexamethasone and the sustained-release OTO-104. Outcomes were synthesized narratively, and the certainty of evidence was assessed using the GRADE framework.

**Results:**

Evidence for the primary outcome—vertigo control—was rated as low certainty, due to methodological heterogeneity, inconsistent findings, and imprecision. Two trials demonstrated significant improvements with ITC compared to placebo, but results were not consistent across studies. Evidence for secondary outcomes, including tinnitus and aural pressure, was of very low to low certainty. Safety outcomes were supported by moderate-certainty evidence, with no serious adverse events reported across studies.

**Conclusions:**

This review highlights the persistent uncertainty surrounding the clinical effectiveness of ITCs in MD. While some evidence supports modest benefit for vertigo control, inconsistencies and low certainty limit generalizability. Future RCTs should prioritize standardized outcome measures and robust methodological design to better define the therapeutic role of ITCs in Menière´s disease.MD.

## Introduction

Ménière’s disease (MD) is a chronic inner ear disorder characterized by spontaneous, episodic attacks of vertigo, fluctuating and progressive sensorineural hearing loss (SNHL), tinnitus, and aural fullness. Both the episodic nature of vertigo and the unpredictable progression of the disease significantly impair patients’ quality of life [1]. The reported global prevalence of MD varies widely, ranging from 3 to 513 cases per 100,000 individuals, reflecting substantial differences across geographic, demographic, and diagnostic contexts [2]. The condition imposes a considerable burden, not only through direct medical costs but also through indirect economic impacts such as reduced work productivity and income loss [3].

Diagnosis is predominantly clinical, guided by patient history and in accordance with the 2015 consensus criteria established by the Bárány Society. MD is recognized as a multifactorial disease, with possible contributions from genetic, autoimmune, allergic, hormonal, and infectious mechanisms [2, 4]. Symptom control primarily targets the management of acute vertigo episodes and prevention of future relapses. Common pharmacologic interventions for acute attacks include benzodiazepines, H1 antihistamines, antidopaminergic agents, anticholinergics, 5-HT3 receptor antagonists, and systemic corticosteroids [5, 6].

In cases refractory to systemic therapy, intratympanic treatments are considered. Intratympanic corticosteroids (ITCs) are often preferred due to their localized anti-inflammatory and vasodilatory effects, with a favorable safety profile marked by minimal risk of hearing deterioration or persistent imbalance. Conversely, intratympanic gentamicin (ITG)-a vestibulotoxic antibiotic used to achieve partial or complete vestibular ablation—is generally reserved for treatment-resistant cases or cases with a severe senosrineural hearing loss. Despite its efficacy in vertigo control, ITG carries a substantial risk of permanent hearing loss and is categorized as a destructive procedure in most clinical guidelines [5, 7, 8]. Corticosteroids are well-established for their anti-inflammatory and immunosuppressive properties. While direct evidence supporting these effects within the inner ear remains limited, pharmacokinetic studies have demonstrated that intratympanic (IT) administration results in significantly higher drug concentrations in the inner ear compared to systemic delivery [9]. Additionally, animal models have reported an increase in cochlear blood flow following IT corticosteroid administration [10]. Some researchers have also hypothesized that corticosteroids may influence salt and fluid metabolism in the inner ear, although current evidence remains inconclusive [7, 11]. Collectively, these findings provide a theoretical rationale for the use of IT corticosteroids (ITC) in treating MD, though further investigation is needed to fully elucidate their mechanisms of action.

Different corticosteroid formulations exhibit varying pharmacokinetic properties, and some may lead to suboptimal drug distribution in critical inner ear structures [12]. Traditional ITCs often offer only temporary symptom relief, necessitating repeated injections. To address these limitations, researchers have investigated sustained-release drug delivery systems—such as thermosensitive hydrogels like OTO-104—to improve inner ear drug residence time and therapeutic efficacy [13].

Reported side effects of ITC therapy include injection-related discomfort, persistent tympanic membrane perforation, and, less frequently, tinnitus, vertigo exacerbation, or hearing loss [14, 16,]. Earlier non-randomized studies already suggested potential benefits of ITCs [17, 18,15], though their methodological limitations highlighted the need for placebo-controlled RCTs.

Commonly used corticosteroids in IT therapy include dexamethasone phosphate, dexamethasone, methylprednisolone succinate, and triamcinolone acetonide. However, there is currently no consensus on the most effective formulation or dosing strategy for MD [14].

## Material end methods

This systematic review was conducted in accordance with the PRISMA 2020 Statement guidelines [19].

Eligible studies were randomized controlled trials (RCTs) that fulfilled the following criteria:

- The intervention involved intratympanic corticosteroids (ITC) compared with placebo.
- Participants were adults (≥ 18 years old).
- No prior surgical treatment for MD had been performed.
- The study was published in English.
- A minimum follow-up duration of 3 months was required, due to the fluctuating and episodic nature of MD.

We searched PubMed, ComDisDome, ScienceDirect, Cochrane Library, Scopus, ClinicalTrials.gov, and the EU Clinical Trials Register from 1995 to 2024 using MeSH terms and free-text keywords related to Ménière’s disease and corticosteroids. Initially, searches were limited to studies from 2010 to 2024, but this was extended to 1995 due to the limited number of RCTs available. Reference lists of relevant reviews and primary studies were also hand-searched to identify additional eligible trials.

Titles and abstracts were screened against the predefined inclusion criteria. Full texts were retrieved for studies that met inclusion criteria or where eligibility remained uncertain. Final eligibility was confirmed after full-text assessment. Any discrepancies were resolved by referring to the inclusion criteria. No automation tools were used in this process.

A standardized data extraction form was developed to collect the following study-level data:

- Study design.
- Population characteristics and sample size.
- Intervention details (corticosteroid type, dose, frequency, number of injections).
- Follow-up duration.
- Primary and secondary outcomes.
- Main results and statistical significance.

The primary outcome was vertigo improvement, assessed through any reported metric including vertigo frequency, vertigo rate, or 28-day Definite Vertigo Days (DVD) reduction from baseline. When the primary outcome was not explicitly stated, it was inferred based on emphasis in the results or discussion sections. For instance, in Garduño-Anaya et al. [16], vertigo improvement assessed by the Class A–F scale was considered primary.

Secondary outcomes included:

- Clinical: tinnitus, aural pressure, quality of life.
- Safety: hearing changes, tympanic membrane perforation, and other adverse events.

Due to heterogeneity in outcome definitions and reporting, secondary outcomes were described narratively.

The Revised Cochrane Risk of Bias Tool for Randomized Trials (RoB 2.0) [20] was used to evaluate five domains:

1. Bias from randomization.
2. Deviations from intended interventions.
3. Missing outcome data.
4. Outcome measurement bias.
5. Selective reporting.

Each study was categorized as having low risk, some concerns, or high risk of bias. No automation tools were used.

Due to substantial heterogeneity in outcome measures, no meta-analysis was conducted. Effect sizes—such as mean differences and proportions—were reported in their original form from each study.

Findings were synthesized narratively under five outcome domains:

1. Vertigo.
2. Tinnitus.
3. Aural pressure.
4. Audiological outcomes.
5. Safety.

Variability in measurement tools and outcomes was addressed in the narrative synthesis.

Potential reporting biases, such as publication bias and selective outcome reporting, were considered during interpretation of the findings.

The GRADE framework (Grading of Recommendations, Assessment, Development and Evaluation) [21] was employed to assess the certainty of the evidence for each outcome, using the following domains:

- Risk of bias.
- Inconsistency.
- Indirectness.
- Imprecision.
- Publication bias.

Each outcome was assigned a certainty level: high, moderate, low, or very low.

## Results

An initial search identified 100 studies. After removing duplicates and conducting title and abstract screening, 16 full-text articles were assessed for eligibility. Of these, 10 studies were excluded.

**Fig. 1:**
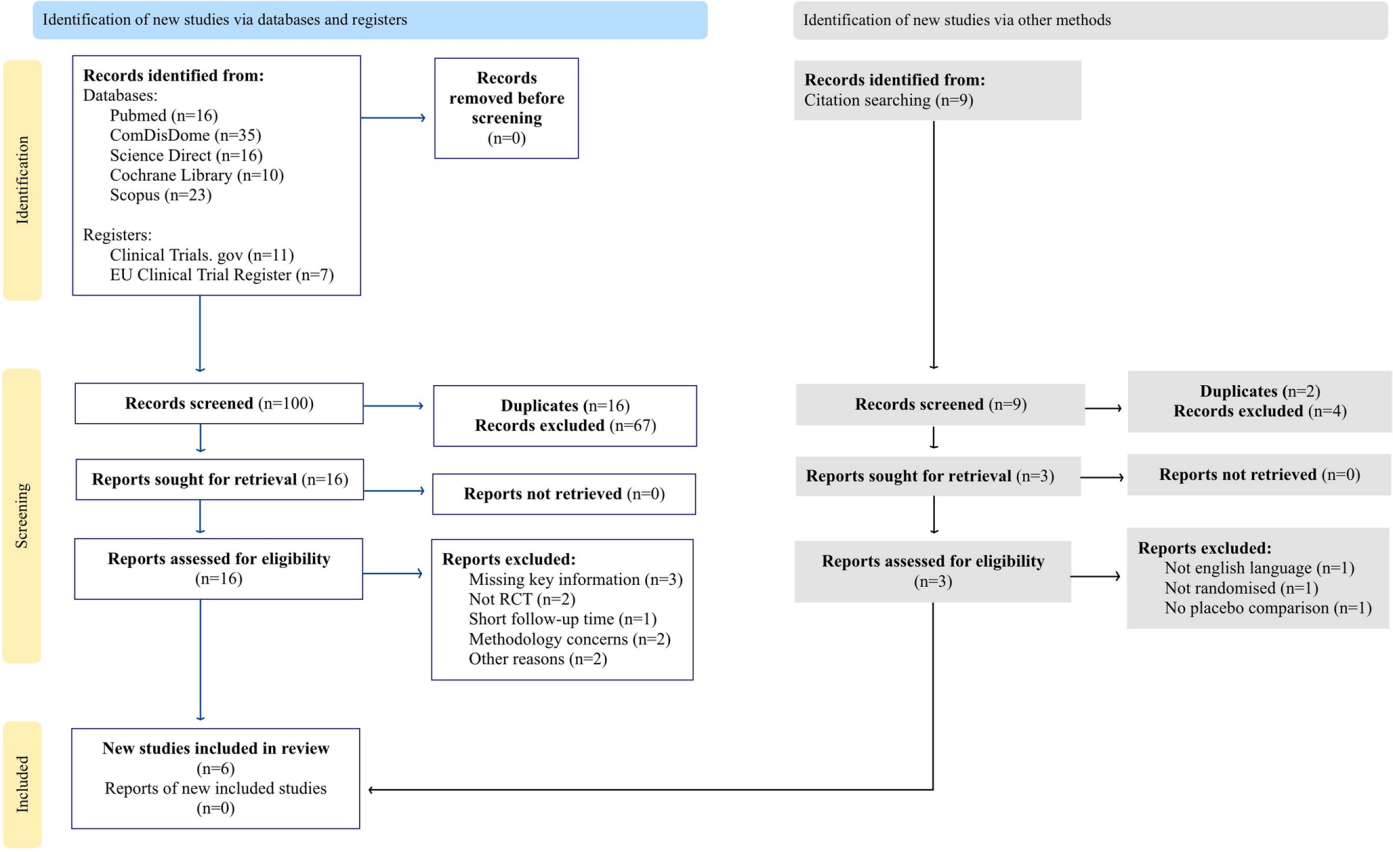
Flow chart of study retrieval and selection

An additional 3 full-text records identified through citation tracking did not meet inclusion criteria, while 2 were duplicates.Thus, 6 RCTs were included in this review:

A PRISMA flow diagram summarizing the selection process is provided in Fig. 1.

### Study characteristics

All six studies were RCTs comparing intratympanic corticosteroids (ITCs**)—**specifically dexamethasone or OTO-104 (a sustained-release formulation)—with placebo (saline or vehicle solution). Follow-up durations ranged from 3 months to 2 years. All trials assessed vertigo as the primary outcome, with secondary outcomes including tinnitus, aural pressure, audiological parameters, quality of life, and safety (See Table 1).

**Table 1:** Vertigo-related outcomes

Study	Outcome Measure	Intervention Group (Mean ± SD if available)	Control Group (Mean ± SD)	*P*-value
**Garduño-Anaya et al.**^16*^ Dexamethasone	**Functional Class (A-F)**	Class A (Complete control) 9/11 (82%) Class B (Substantial control) 2/11 (18%)	Class A: 4/7 (57%) Class B: 0/7 Class F (Failure): 3/7 (43%)	*p* < 0.001
(4 mg/mL x 0.5 mL x 4 consecutive days)	**Functional Level Scale (proportion of Level 1)**	10/11 (91%)	3/7 (43%)	*p* < 0.001
	DHI Score improvement	60.4	41.3	*p* < 0.008
	Subjective Vertigo Improvement Scale (0–10)	9/10 (90%)	5.7/10 (57%)	*p* < 0.001
**Lambert et al.** ^27^** **Low-dose cohort**	**Change in Vertigo Frequency**	−0.147 ± 0.166	−0.124 ± 0.153	*p* = 0.493
3 mg OTO-104 (Single injection)	Vertigo Severity Score	−602 ± 0.539	−0.467 ± 0.476	Not provided
Lambert et al.^27^>** **High-dose cohort**	**Change in Vertigo Frequency**	−0.211 ± 0.153	−0.124 ± 0.153	*p* = 0.086
12 mg OTO-104 (Single injection)	Vertigo Severity Score	−0.589 ± 0.321	−0.467 ± 0.476	Not provided
**Lambert et al.** ^13**^	**Change in vertigo rate**	−4.6 (61%)	−3.2 (43%)	*p* = 0.067
12 mg OTO-104 (Single injection)	Number of Definitive Vertigo Days	1.64	2.54	*p* = 0.03
	Vertigo Severity Score	−0.46	−0.32	*p* = 0.046
	Average daily vertigo count	−0.53	−0.33	*p* = 0.065
**AVERTS-1**^24**^	**28-Day Average DVD**	2.191 (1.644 to 2.919)	2.415 (1.823 to 3.198)	*p* = 0.623
12 mg OTO-104 (Single injection)	Mean Vertigo Severity Score	0.408 ± 0.046	0.403 ± 0.046	*p* = 0.932
	Change in Vertigo Frequency	−0.173 ± 0.018	−0.173 ± 0.018	*p* = 0.993
	Number of sick days or bedridden	1.287 ± 2.879	1.058 ± 2.077	*p* = 0.943
**AVERTS-2 (FAS-2)** ^25**^	**28-Day Average DVD**	2.044 (1.480 to 2.822)	3.467 (2.617 to 4.594)	*p* = 0.014
12 mg OTO-104 (Single injection)	Mean Vertigo Severity Score	0.400	0.583	*p* = 0.030
	Change in Vertigo Frequency	−0.205	−0.128	*p* = 0.030
	Number of sick days or bedridden	0.627	1.179	*p* = 0.028
**811 Trial** ^26**^	**28-Day Average DVD**	2.869 (2.109 to 3.903)	3.577 (2.641 to 4.844)	*p* = 0.312
12 mg OTO-104 (Single injection)	Mean Vertigo Severity Score	0.425 ± 0.052	0.440 ± 0.051	*p* = 0.832
	Change in Vertigo Frequency	−0.207 ± 0.021	−0.176 ± 0.021	*p* = 0.282
	Number of sick days or bedridden	0.859	1.563	*p* = 0.053

*Follow-up time: 2 years; ** Follow-up time: 3 months. Primary outcome of each study appears in bold

#### Note

The three large OTO-104 trials reported by Phillips et al. in 2023 [22] and Scarpa et al. [] in 2024 correspond to the AVERTS-1 [24], AVERTS-2 [25], and 811 [26] studies, all of which were included in this review. AVERTS trials excluded recent ITC or steroid users, while the 811 trial excluded those with any ITC history.

### Risk of bias in included studies

Using the RoB 2.0 tool [20], most studies showed low risk for blinding and outcome measurement and some concerns regarding randomization and missing data handling. Methods for sequence generation and allocation concealment were often not explicitly described, and dropout handling lacked transparency. A summary is available in Table 2.

**Table 2:** Risk of bias summary

Study	Randomization process	Deviations from intended interventions	Missing outcome data	Measurement of outcome	Selection of reported result	Overall risk of bias
Garduño-Anaya et al.^16^	Some concerns	Low risk	Some concerns	Low risk	Low risk	**Some concerns**
Lambert et al.^13^	Some concerns	Low risk	Some concerns	Low risk	Low risk	**Some concerns**
Lambert et al.^27^	Some concerns	Low risk	Low risk	Some concerns	Low risk	**Some concerns**
AVERTS-1^24^	Some concerns	Low risk	Some concerns	Low risk	Low risk	**Some concerns**
AVERTS-2^25^	Some concerns	Low risk	Some concerns	Low risk	Some concerns	**Some concerns**
Study 811^26^	Some concerns	Low risk	Low risk	Low risk	Low risk	**Low risk**

### Results of individual studies and narrative synthesis

#### Vertigo (Primary Outcome)

Only two trials demonstrated statistically significant superiority of ITC over placebo for vertigo control [16, 23]. However, comparison across studies was hampered by:

- Follow-up variation: e.g., Garduño-Anaya [16] (2 years) vs. others (3 months).
- Formulation differences: sustained-release OTO-104 vs. conventional dexamethasone.
- Measurement inconsistency: vertigo days, rates, severity scales—all varied across trials.

Among the six RCTs included in the review, outcomes related to vertigo varied in both measurement and statistical significance. Vertigo control was the primary endpoint in all trials, assessed via diverse metrics such as vertigo frequency, rate, definitive vertigo days (DVD), or severity scores.

The study by Garduño-Anaya et al. [16] showed the most robust results, reporting statistically significant improvements in multiple vertigo-related measures over a two-year period. In the treatment group, 82% achieved complete vertigo control (Class A), compared to 57% in the placebo group, with a p-value < 0.001. Dizziness Handicap Inventory (DHI) scores improved by a mean of 60.4 points vs. 41.3 in controls (*p* = 0.008), and subjective vertigo improvement reached 90% in the treated group (*p* < 0.001).

In contrast, most trials using OTO-104 with a single 12 mg intratympanic injection over 3 months demonstrated less conclusive findings. For example:

- Lambert et al. (2016) [13] showed a reduction in vertigo rate by 4.6 episodes vs. 3.2 in placebo (*p* = 0.067), with only some endpoints achieving statistical significance such as DVD (*p* = 0.03) and vertigo severity score (*p* = 0.046). Earlier, Lambert et al. (2012) [27] reported results from a smaller randomized trial of a single OTO-104 injection, showing trends toward improvement but without consistent statistical significance across endpoints. These preliminary findings preceded the larger phase 2b and phase 3 studies.
- AVERTS-2 [25], one of the few studies with significant outcomes, reported a reduction in average DVD to 2.04 from 3.47 in placebo (*p* = 0.014), along with improvements in severity (*p* = 0.030) and frequency (*p* = 0.030).
- AVERTS-1 [24] and the 811 trial [26], however, failed to demonstrate statistically significant differences between ITC and placebo for primary vertigo outcomes.

Vertigo severity and daily count showed numerical improvement in some trials but did not consistently reach significance thresholds. For instance, in the 811 trial [26], average DVD and vertigo severity score changes trended in favor of treatment but p-values remained > 0.3.

Overall, while certain trials—particularly Garduño-Anaya et al. [16] and AVERTS-2 [25] showed statistically significant benefits of ITC on vertigo outcomes, most others reported non-significant or borderline results. The findings suggest potential but inconsistent efficacy, emphasizing the need for larger, standardized trials with harmonized outcome measures.

See see Table 3 for overall results.

**Table 3:** Vertigo-related outcomes. *Follow-up time: 2 years; ** Follow-up time: 3 months. Primary outcome of each study appears in bold

Study	Outcome Measure	Intervention Group (Mean ± SD if available)	Control Group (Mean ± SD)	*P*-value
**Garduño-Anaya et al.**^16*^ Dexamethasone	**Functional Class (A-F)**	Class A (Complete control) 9/11 (82%) Class B (Substantial control) 2/11 (18%)	Class A: 4/7 (57%) Class B: 0/7 Class F (Failure): 3/7 (43%)	*p* < 0.001
(4 mg/mL x 0.5 mL x 4 consecutive days)	**Functional Level Scale (proportion of Level 1)**	10/11 (91%)	3/7 (43%)	*p* < 0.001
	DHI Score improvement	60.4	41.3	*p* < 0.008
	Subjective Vertigo Improvement Scale (0–10)	9/10 (90%)	5.7/10 (57%)	*p* < 0.001
**Lambert et al.** ^27^** **Low-dose cohort**	**Change in Vertigo Frequency**	−0.147 ± 0.166	−0.124 ± 0.153	*p* = 0.493
3 mg OTO-104 (Single injection)	Vertigo Severity Score	−602 ± 0.539	−0.467 ± 0.476	Not provided
**Lambert et al.**^27^** **High-dose cohort**	**Change in Vertigo Frequency**	−0.211 ± 0.153	−0.124 ± 0.153	*p* = 0.086
12 mg OTO-104 (Single injection)	Vertigo Severity Score	−0.589 ± 0.321	−0.467 ± 0.476	Not provided
**Lambert et al.** ^13**^	**Change in vertigo rate**	−4.6 (61%)	−3.2 (43%)	*p* = 0.067
12 mg OTO-104 (Single injection)	Number of Definitive Vertigo Days	1.64	2.54	*p* = 0.03
	Vertigo Severity Score	−0.46	−0.32	*p* = 0.046
	Average daily vertigo count	−0.53	−0.33	*p* = 0.065
**AVERTS-1**^24**^	**28-Day Average DVD**	2.191 (1.644 to 2.919)	2.415 (1.823 to 3.198)	*p* = 0.623
12 mg OTO-104 (Single injection)	Mean Vertigo Severity Score	0.408 ± 0.046	0.403 ± 0.046	*p* = 0.932
	Change in Vertigo Frequency	−0.173 ± 0.018	−0.173 ± 0.018	*p* = 0.993
	Number of sick days or bedridden	1.287 ± 2.879	1.058 ± 2.077	*p* = 0.943
**AVERTS-2 (FAS-2)** ^25**^	**28-Day Average DVD**	2.044 (1.480 to 2.822)	3.467 (2.617 to 4.594)	*p* = 0.014
12 mg OTO-104 (Single injection)	Mean Vertigo Severity Score	0.400	0.583	*p* = 0.030
	Change in Vertigo Frequency	−0.205	−0.128	*p* = 0.030
	Number of sick days or bedridden	0.627	1.179	*p* = 0.028
**811 Trial** ^26**^	**28-Day Average DVD**	2.869 (2.109 to 3.903)	3.577 (2.641 to 4.844)	*p* = 0.312
12 mg OTO-104 (Single injection)	Mean Vertigo Severity Score	0.425 ± 0.052	0.440 ± 0.051	*p* = 0.832
	Change in Vertigo Frequency	−0.207 ± 0.021	−0.176 ± 0.021	*p* = 0.282
	Number of sick days or bedridden	0.859	1.563	*p* = 0.053

### Secondary outcomes

#### Tinnitus

Three trials assessed tinnitus [14, 16, 25], but none reported statistically significant differences between ITC and placebo.

#### Aural pressure

Only Garduño-Anaya et al. [16] measured this outcome, reporting significant improvement with ITC (48.1% vs. 20%, *p* = 0.005).

#### Quality of life

Five studies included quality-of-life measures, but none found meaningful between-group differences.

#### Safety

No serious adverse events were reported across studies. Tympanic membrane perforation was rare and occurred at similar rates in both groups. No worsening in hearing thresholds was noted in treatment vs. placebo arms.Adverse events (e.g., injection site pain, transient dizziness) were mild and self-limiting.

## Discussion

The findings of this systematic review are consistent with the conclusions of Basura et al. [8], whose clinical practice guideline on MD categorizes treatment interventions as “strong recommendations,” “recommendations,” or “options.” Intratympanic corticosteroids (ITCs) are categorized as an “option”, reflecting either low-quality evidence (Grade D) or a lack of compelling evidence favoring one treatment over another. This classification parallels our findings, which underscore a low certainty of evidence supporting ITCs for MD. Our conclusions are consistent with earlier systematic reviews, including Devantier et al. [28], and the recent scoping review by Oscé et al. [29], both of which emphasized the need for standardized outcomes and more rigorous trial design.

Similarly, the systematic review by Webster et al. [15] also highlights the considerable uncertainty surrounding the efficacy of ITCs. Like our findings, they observed that while some studies report modest improvements in vertigo control, these benefits are not consistent across trials or statistically significant. The overlap in studies included in both reviews reinforces the reliability of these findings while also emphasizing the scarcity of high-quality RCTs available on this topic.

Duration of disease history is a critical prognostic factor: several studies have shown that vertigo attacks may remit spontaneously over time [30–32]. This natural course complicates interpretation of intervention trials, since spontaneous improvement may be misattributed to treatment effect.

A major limitation of the current evidence lies in the heterogeneity of study designs, formulations, and outcome measures. Although all included studies were RCTs, their methodological diversity—ranging from differing ITC formulations (e.g., sustained-release vs. standard dexamethasone), dosing schedules, and follow-up durations—precluded direct comparison and robust synthesis. This variability significantly undermines the ability to draw generalizable conclusions.

The limited number of placebo-controlled RCTs further weakens the evidence base. However, ongoing trials such as NCT05851508 [33], identified during our search, may address these shortcomings by implementing standardized outcomes and rigorous methodology, offering future opportunities for higher-quality synthesis.

Several potentially relevant studies were excluded due to insufficient reporting. For instance, El-Shafei et al. [34] lacked a placebo arm, and Borghei et al. [35] did not report essential protocol elements such as dose and frequency, precluding meaningful inclusion. Improved transparency and completeness in trial reporting would greatly enhance the value of future evidence. Furthermore, Yoda et al. [36] observed a false membrane covering the round window niche in approximately 50% of cases, potentially impeding drug diffusion. This anatomical barrier may contribute to the variability in clinical responses to ITCs and underscores the need to consider middle and inner ear anatomy when evaluating treatment efficacy.

Lastly, the lack of uniform outcome measures—both primary and secondary—makes cross-study comparisons challenging. The use of diverse metrics (e.g., vertigo days, frequency, subjective improvement scales) adds to the overall uncertainty.

Our findings largely confirm previous systematic reviews [8, 15], and while this limits the novelty of the present work, it reinforces the consistency of evidence showing uncertainty about the efficacy of ITC in Ménière’s disease.

Despite a comprehensive and sensitive search strategy, this review may still be subject to publication bias, particularly due to the exclusion of non-English and unpublished studies. The narrative synthesis approach—adopted due to heterogeneity—lacks the statistical rigor of meta-analysis and may introduce interpretation bias. Future systematic reviews would benefit from more homogeneous study designs and greater transparency in reporting, which would permit robust meta-analytical techniques.

Future RCTs should adopt multicenter, double-blind designs with standardized outcome measures, ≥ 12 months follow-up, and stratification by disease duration. Use of common endpoints such as Definite Vertigo Days and DHI scores would allow robust meta-analysis. Pragmatic trial designs with patient-centered outcomes are feasible and urgently needed.

Another limitation is the lack of systematic assessment of psychiatric comorbidity, which is increasingly recognized to affect MD outcomes. A recent meta-analysis demonstrated significant associations of MD with depression and anxiety [37], suggesting that comorbidity may influence both treatment response and quality-of-life outcomes.

## Conclusions

This systematic review highlights the persistent uncertainty in the evidence supporting the use of intratympanic corticosteroids for MD. Although some studies report modest benefits for vertigo control, especially in long-term or sustained-release formulations, the overall quality and consistency of the evidence remain low. Major limitations include a limited number of RCTs, variation in formulations and dosages, and inconsistent outcome definitions. To strengthen the clinical evidence base, future studies must prioritize well-designed, placebo-controlled RCTs that employ standardized interventions and outcome measures. Harmonizing follow-up durations, improving anatomical targeting, and incorporating patient-centered endpoints will also enhance evidence quality. Importantly, enabling a future meta-analysis will depend on achieving greater methodological uniformity across trials. Enhanced data transparency and thorough reporting of protocols and outcomes are essential for improving both research reproducibility and clinical applicability.

## References

[1] Gürkov R, Pyykö I, Zou J, Kentala E (2016) What is Menière’s disease? A contemporary re-evaluation of endolymphatic hydrops. J Neurol 263(Suppl 1):S71–81. 10.1007/s00415-015-7930-1 27083887 10.1007/s00415-015-7930-1 PMC4833790

[2] Tyrrell J, Whinney DJ, Taylor T (2016) The cost of Ménière’s disease: a novel multisource approach. Ear Hear 37(3):e202–e209. 10.1097/AUD.0000000000000264 26760200 10.1097/AUD.0000000000000264

[3] Hoskin JL (2022) Ménière’s disease: new guidelines, subtypes, imaging, and more. Curr Opin Neurol 35(1):90–97. 10.1097/WCO.0000000000001021 34864755 10.1097/WCO.0000000000001021

[4] Frejo L, Martin-Sanz E, Teggi R et al (2017) Extended phenotype and clinical subgroups in unilateral Meniere disease: A cross‐sectional study with cluster analysis. Clin Otolaryngol 42(6):1172–1180. 10.1111/coa.12844 28166395 10.1111/coa.12844

[5] Perez-Carpena P, Lopez-Escamez JA (2020) Current Understanding and clinical management of Menière’s disease: a systematic review. Semin Neurol 40(1):138–150. 10.1055/s-0039-3402065 31887752 10.1055/s-0039-3402065

[6] Webster KE, Galbraith K, Harrington-Benton NA (2023) Systemic pharmacological interventions for Ménière’s disease. Cochrane Database Syst Rev (2):CD015171. 10.1002/14651858.CD015171.pub2 10.1002/14651858.CD015171.pub2 PMC9948543 36827524

[7] Pullens B, van Benthem PP (2011) Intratympanic gentamicin for Ménière’s disease or syndrome. Cochrane Database Syst Rev 3CD008234. 10.1002/14651858.CD008234.pub2 10.1002/14651858.CD008234.pub2 PMC13378876 21412917

[8] Basura GJ, Adams ME, Monfared A et al (2020) Clinical practice guideline: Ménière’s disease executive summary. Otolaryngol Head Neck Surg 162(4):415–434. 10.1177/0194599820909439 32267820 10.1177/0194599820909439

[9] Salt AN, Plontke SK (2018) Pharmacokinetic principles in the inner ear: influence of drug properties on intratympanic applications. Hear Res 368:28–40. 10.1016/j.heares.2018.03.002 29551306 10.1016/j.heares.2018.03.002 PMC6133771

[10] Chi FL, Yang MQ, Zhou YD, Wang B (2011) Therapeutic efficacy of topical application of dexamethasone to the round window niche after acoustic trauma caused by intensive impulse noise in Guinea pigs. J Laryngol Otol 125(7):673–685. 10.1017/S0022215111000028 21693072 10.1017/S0022215111000028

[11] Bogaz EA, Maranhão AS, Inoue DP et al (2017) Ménière’s disease treatment. Int Arch Otorhinolaryngol 21(2):184–191. 10.1055/s-0036-158352428382129

[12] Salt AN, Hartsock JJ, Hou J, Piu F (2019) Comparison of the pharmacokinetic properties of triamcinolone and dexamethasone for local therapy of the inner ear. Front Cell Neurosci 13:347. 10.3389/fncel.2019.00347 31427927 10.3389/fncel.2019.00347 PMC6689996

[13] Lambert PR, Carey J, Mikulec AA et al (2016) Intratympanic sustained-exposure dexamethasone thermosensitive gel: randomized phase 2b trial. Otol Neurotol 37(10):1669–1676. 10.1097/MAO.0000000000001227 27749754 10.1097/MAO.0000000000001227 PMC5414596

[14] Li S, Pyykkö I, Zhang Q et al (2022) Consensus on intratympanic drug delivery for Menière’s disease. Eur Arch Otorhinolaryngol 279(8):3795–3799. 10.1007/s00405-022-07374-y 35469039 10.1007/s00405-022-07374-y PMC9249695

[15] Webster KE, Lee A, Galbraith K et al (2023) Intratympanic corticosteroids for Ménière’s disease. Cochrane Database Syst Rev (2):CD015245. 10.1002/14651858.CD015245.pub2 10.1002/14651858.CD015245.pub2 PMC9969957 36847608

[16] Garduño-Anaya MA, De Couthino H et al (2005) Dexamethasone inner ear perfusion by intratympanic injection in unilateral Ménière’s disease: a two-year prospective trial. Otolaryngol Head Neck Surg 133(2):285–294. 10.1016/j.otohns.2005.05.010 16087029 10.1016/j.otohns.2005.05.010

[17] Boleas-Aguirre MS, Lin FR, Della Santina CC, Minor LB, Carey JP (2008) Longitudinal results with intratympanic dexamethasone in the treatment of Ménière’s disease. Otol Neurotol 29(1):33–38. 10.1097/mao.0b013e31815dbafc 18199956 10.1097/mao.0b013e31815dbafc PMC2937266

[18] Herraiz C, Plaza G, Aparicio JM, Gallego I, Marcos S, Ruiz C (2010) Transtympanic steroids for Ménière’s disease. Otol Neurotol. 31(1):162–167. 10.1097/MAO.0b013e3181c34e53 19924013 10.1097/MAO.0b013e3181c34e53

[19] Page MJ, McKenzie JE, Bossuyt PM, Boutron I, Hoffmann TC, Mulrow CD, et al. The PRISMA 2020 statement: an updated guideline for reporting systematic reviews. BMJ. 2021;372:n71. doi: 10.1136/bmj.n71 10.1136/bmj.n71 PMC8005924 33782057

[20] Sterne JAC, Savović J, Page MJ, Elbers RG, Blencowe NS, Boutron I, et al. RoB 2: a revised tool for assessing risk of bias in randomised trials. BMJ. 2019;366:l4898. doi: 10.1136/bmj.l4898 10.1136/bmj.l4898 31462531

[21] Guyatt GH, Oxman AD, Vist GE, Kunz R, Falck-Ytter Y, Alonso-Coello P, Schünemann HJ; GRADE Working Group. GRADE: an emerging consensus on rating quality of evidence and strength of recommendations. BMJ. 2008;336(7650):924–926. doi: 10.1136/bmj.39489.470347. 10.1136/bmj.39489.470347.AD PMC2335261 18436948

[22] Phillips J, Mikulec AA, Robinson JM, Skarinsky D, Anderson JJ (2023) Efficacy of intratympanic OTO-104 for the treatment of Ménière’s disease: outcome of three randomized, double-blind, placebo-controlled studies. Otol Neurotol 44(6):584–592. 10.1097/MAO.0000000000003886 37185596 10.1097/MAO.0000000000003886 PMC10289225

[23] Scarpa A, Carucci M, Ralli M, De Luca P, Salzano G, Viola P, Chiarella G, Salzano FA (2024) Efficacy of intratympanic OTO-104 for the treatment of Ménière’s disease: outcome of three randomized, double-blind, placebo-controlled studies. Otol Neurotol 45(9):1087–1088. 10.1097/MAO.0000000000004289 39186046 10.1097/MAO.0000000000004289

[24] AVERTS-1 Study of OTO-104 in subjects with unilateral Menière’s disease (2023) ClinicalTrials Gov NCT02612337

[25] AVERTS-2 Study of OTO-104 in subjects with unilateral Menière’s disease (2023) ClinicalTrials Gov NCT02717442

[26] Clinical Study 811 Phase 3 Study of OTO-104 in Subjects With Unilateral Menière’s Disease (2022) ClinicalTrials Gov NCT03664674

[27] Lambert PR, Nguyen S, Maxwell KS et al (2012) A randomized, double-blind, placebo-controlled clinical study to assess safety and clinical activity of OTO-104 given as a single intratympanic injection. Otol Neurotol 33(7):1257–1265. 10.1097/MAO.0b013e318263d35d 22858715 10.1097/MAO.0b013e318263d35d

[28] Devantier L, Djurhuus BD, Hougaard DD, Händel MN, Guldfred FL, Schmidt JH, Edemann-Callesen H (2019) Intratympanic steroid for Ménière’s disease: a systematic review. Otol Neurotol 40(6):806–812. 10.1097/MAO.0000000000002255 31135678 10.1097/MAO.0000000000002255

[29] Oscé H, Loos E, Huygen A, Desloovere C (2025) Treatment of Ménière’s disease: a scoping review of the current evidence. Eur Arch Otorhinolaryngol 282(8):3897–3923. 10.1007/s00405-025-09329-5 40204985 10.1007/s00405-025-09329-5

[30] Gerritsen FR, Schenck AA, Locher H, van de Berg R, van Benthem PP, Blom HM (2024) The evolution of intractable Ménière’s disease: attacks resolve over time. Front Neurol 15:1469276. 10.3389/fneur.2024.1469276 39512276 10.3389/fneur.2024.1469276 PMC11542254

[31] Pyykko I, Zou J, Vetkas N (2024) Changes in symptom pattern in Meniere’s disease by duration: the need for comprehensive management. Front Neurol 15:1496384. 10.3389/fneur.2024.1496384 39582681 10.3389/fneur.2024.1496384 PMC11581947

[32] Sumi T, Watanabe I, Tsunoda A, Nishio A, Komatsuzaki A, Kitamura K. Longitudinal study of 29 patients with Ménière’s disease with follow-up of 10 years or more. Acta Otolaryngol. ;132:10–15. doi:10.3109/00016489.2011.627570 10.3109/00016489.2011.627570 22054051

[33] Boreel EM, van Esch B, Schermer TR, Mol BM, van Benthem P, Bruintjes TD. The effectiveness of intratympanic injections with methylprednisolone versus placebo in the treatment of vertigo attacks in Ménière’s disease (PREDMEN trial): a study protocol for a phase-3 multicentre, double-blinded, randomised, placebo-controlled trial. BMJ Open. 2024;14(8):e076872. doi: 10.1136/bmjopen-2023-076872 10.1136/bmjopen-2023-076872 PMC11367374 39209781

[34] El Shafei RR, Qotb M (2020) Comparison of the effect of three treatment interventions for the control of Menière’s disease: a randomized control trial. Egypt J Otolaryngol 36:22

[35] Borghei P, Sadeghian E, Hasanzadeh F, Emami H. Intratympanic dexamethasone delivery versus placebo in intractable Menière disease. AJS. ;3(3–4):58–62.

[36] Yoda S, Cureoglu S, Shimizu S, Morita N, Fukushima H, Sato T, Harada T, Paparella MM (2011) Round window membrane in Ménière’s disease: a human temporal bone study. Otol Neurotol. 32(1):147–151. 10.1097/MAO.0b013e318200a0e0 21131881 10.1097/MAO.0b013e318200a0e0

[37] Yeo BSY, Toh EMS, Lim NE, Lee RS, Ho RCM, Tam WWS, Ngo RYS (2025) Association of Menière’s disease with depression and anxiety: a systematic review and meta-analysis. Eur Arch Otorhinolaryngol. 10.1007/s00405-025-09297-w 40087158 10.1007/s00405-025-09297-w

